# Assessment of the Relationship Between ABO Blood Group and Familial Cancer Occurrence in Kirkuk City

**DOI:** 10.31661/gmj.v14i.3813

**Published:** 2025-03-17

**Authors:** Sunbul Mohammed Zeki Abdullah, Mayada K. Mohammed, Maha A. Hamdi

**Affiliations:** ^1^ Department of Family and Community Medicine, Tikrit Medical College, Tikrit University, Iraq

**Keywords:** ABO Blood Groups, Cancer, Iraq, Familial Cancer

## Abstract

**Background:**

The potential connection between blood types and cancer risk has become a puzzling yet intriguing area of research in recent years; while its association is widely assessed with cancer occurrence, it is not well assessed in case of hereditary cancers. This study aimed to assess the relation between ABO blood groups and the occurrence of familial cancers among the patients with cancer in Kirkuk city, Iraq.

**Materials and Methods:**

This cross-sectional study was carried out in Kirkuk city from the period 15th October 2023 to 15th April 2024 on 398 patients diagnosed with cancer in Kirkuk oncology center. The study included three groups: patients with a family history of cancer in first-degree relatives (n=90), no family history (n=288), and second-degree relatives (n=20). Data was collected from both medical records and patient interviews, focusing on demographic details, lifestyle habits, medical history, and exposure to potential risk factors. In-depth interviews provided further details on lifestyle patterns, including smoking habits, alcohol use, eating habits, medical conditions, medication use, viral infections, and exposure to chemicals or radiation. Data was analyzed using SPSS V.26 comparing based on the familial cancer history.

**Results:**

Significant differences were noted in age distribution, with the 38-47 and 48-57 age groups being more common in the first-degree and no family history groups, respectively. Sex distribution showed a higher proportion of females in the first-degree and second-degree groups (P=0.017). There were higher proportions of high socioeconomic status in the second-degree group and married individuals in the first-degree and second-degree groups compared to non-familial cancers. Breast cancer was more prevalent in the first-degree and second-degree groups. Adjusting for demographic, socioeconomic, Body-Mass-Index (BMI), cancer type, and health related habits, O- blood group was significantly associated with a higher likelihood of familial cancer history in first-degree relatives (OR: 14.083, P=0.024) compared to non-familial ones. Other blood groups did not show significant associations.

**Conclusion:**

There might be an association between familial cancer O- blood group that needs to be re-evaluated in further studies.

## Introduction

There are many types of cancer, and some cells grow and spread without being managed. There are still a lot of deaths from it around the world, which is a big problem for public health [1]. Over the past few years, the link between blood group and cancer risk has been getting more and more attention. This is one of many things being studied [2][3]. It was discovered more than fifty years ago by a British research group that people with stomach cancer are more likely to have blood group A and less likely to have blood group O. It was the first study to try to find a link between ABO blood types and the chance of getting cancer, so it was very important. The ABO blood group system is one of the most well-known and studied in people. People have looked at it as a possible sign of cancer risk and result [4][5]. It was Karl Landsteiner who came up with the ABO blood group method in the early 1900s. People are put into four main blood groups based on whether or not antigens (A and B) are on the surface of their red blood cells. The blood groups are O, B, AB, and A. Physicians are very interested in the ABO blood group system, both for how it is used to send blood to different people and for what it might mean for some health problems, like cancer [6]. A lot of genetic and population studies have been sparked by the idea that blood group might affect how likely someone is to get some diseases, like cancer [7]. ABO blood group and solid cancers like pancreatic, stomach, and colon cancers have been studied, but the results are not yet clear [8]. These links aren’t very strong, though, and are usually trumped by stronger risk factors like genes, lifestyle choices, and the environment [9]. It’s also not clear what the link is between the ABO blood group and the risk of colon cancer. One study found that people with blood group A had a slightly higher risk. But these results are still not reliable, and we still don’t fully understand the link. It is thought that solid cancer is caused by other things more than one. As more research is done in this area, we may learn more about the possible links between ABO blood group and solid cancer risk [10]. Notably, ABO blood groups have been linked to a higher chance of cancer through immune response, inflammation, and blood clotting, all of which play a part in how cancer grows and spreads [1]. For instance, a large-scale meta-analysis of over 20 million participants revealed that non-O blood groups, particularly A and AB, are associated with an increased risk of pancreatic cancer in both Caucasians and Asians [11]. Similarly, a systematic review and meta-analysis of individual cancer sites found that blood group A is linked to an elevated risk of gastric, pancreatic, breast, ovarian, and nasopharyngeal cancers [12]. As the current understanding of the relationship between ABO blood groups and familial cancer is limited and inconclusive, with few studies having investigated this specific association, we aimed to assess the relation between ABO blood groups and the occurrence of familial cancers. Also, what makes this study novel is its focus on exploring the potential link between ABO blood groups and familial cancer, which may uncover new insights into the genetic predisposition of cancer in families, particularly in the context of first-degree relatives, and provide a foundation for future research in this understudied area.

## Patients and Methods

This cross-sectional study was carried out in Kirkuk city from the period 15th October 2023 to 15th April 2024 on cancer patients of Kirkuk oncology center. Data was collected from medical records and patient interviews to examine demographic characteristics, lifestyle factors, medical history, and potential exposures. The study included 398 patients diagnosed with cancer within a specified time frame, selected from medical records of healthcare facilities in the target area. The inclusion criteria for this study consisted of patients diagnosed with cancer within a specified time frame, who were registered at the Kirkuk Oncology Center and had complete medical records. Patients were also required to be willing and able to participate in the study, and to provide informed consent. The exclusion criteria included patients who were not diagnosed with cancer, those with incomplete medical records, and those who declined to participate in the study or were unable to provide informed consent. Additionally, patients with a history of cancer treatment or those who had been diagnosed with cancer outside of the specified time frame were also excluded from the study. The sampling method of this study was simple selection in the mentioned timeline to cover all cases.

Participants were categorized based on age, sex, residence, educational level, socioeconomic status, occupation, blood group, Rh factor, cancer type, family history of cancer, marital status, smoking and alcohol consumption habits, body mass index (BMI), dietary habits, medical conditions, drug and medication history, viral infections, and exposures to chemicals or radiation. Patient interviews were conducted to obtain additional details on lifestyle factors such as smoking and alcohol consumption habits, dietary patterns, medical conditions, drug and medication history, viral infections (HIV, hepatitis, HPV), and exposures to chemicals or radiation.

The blood group of the participants was determined through a review of their medical records, which contained information on their blood type and Rh factor. However, for participants whose blood group was not documented in their medical records, laboratory tests were conducted to determine their blood group. A 5ml blood sample was collected from these participants and sent to the laboratory for blood grouping and Rh typing using the standard tube method. The blood samples were tested using anti-A and anti-B antibodies to determine the presence or absence of A and B antigens on the surface of the red blood cells. The results were then recorded and used to categorize the participants into their respective blood groups (A, B, AB, or O) and Rh factor status (Rh positive or Rh negative).

## Ethical approval

Approval of the council of College of Medicine/ Tikrit University was obtained for the proposal of the study. Approval permission was presented to the director of Kirkuk Health Directorate / Kirkuk Oncology Center.

## Statistical Analysis

Descriptive statistics were used to summarize participant characteristics and factors associated with cancer incidence. Statistical analyses, including chi-square tests and regression models, were conducted to assess the relationship between various factors and cancer incidence. Computerized statistically analysis was performed using IBM SPSS Statistics for Windows, version 26 (IBM Corp., Armonk, N.Y., USA). Comparison was carried out using, Chi-square and ANOVA for correlation and for determination of probability value (P-value). To further explore the association between ABO/Rh blood groups and familial cancer history, a multinomial logistic regression analysis was conducted, adjusting for several demographic and lifestyle factors including age, sex, marital status, residence, education, socioeconomic status, occupation, type of cancer, smoking, alcohol consumption, dietary habits, and BMI. The model’s goodness of fit was assessed using the Pearson chi-squared test, and the results showed that the model fit the data well. The P value≥ 0.05 was considered statistically significant.

## Results

**Table:** Table1. Demographic, clinical, and life style related variables of study

**Variable**	**First Degree (n=90)**	**None (n=288)**	**Second Degree (n=20)**	**p-value**	
**Age (year)**					0.052
	**18-27**	1(1.11%)	7(2.43%)	0(0%)	
	**28-37**	4(4.44%)	16(5.56%)	1(5%)	
	**38-47**	16(17.78%)	39(13.54%)	5(25%)	
	**48-57**	33(36.67%)	86(29.86%)	6(30%)	
	**58-67**	28(31.11%)	70(24.31%)	7(35%)	
	**68-77**	6(6.67%)	47(16.32%)	0(0%)	
	**78-87**	2(2.22%)	21(7.29%)	0(0%)	
	**<18**	0(0%)	2(0.69%)	1(5%)	
**Sex**					0.017
	**Female**	71(78.89%)	194(67.36%)	18(90%)	
	**Male**	19(21.11%)	94(32.64%)	2(10%)	
**Residence**			0.268
	**Rural**	24(26.67%)	89(30.9%)	3(15%)	
	**Urban**	66(73.33%)	199(69.1%)	17(85%)	
**Educational Level**					0.614
	**High education**	27(30%)	66(22.92%)	7(35%)	
	**Illiterate**	19(21.11%)	72(25%)	4(20%)	
	**Primary**	30(33.33%)	108(37.5%)	8(40%)	
	**Secondary**	14(15.56%)	42(14.58%)	1(5%)	
**Socioeconomic State**					0.055
	**High**	15(16.67%)	29(10.07%)	6(30%)	
	**Low**	18(20%)	67(23.26%)	5(25%)	
	**Middle**	57(63.33%)	192(66.67%)	9(45%)	
**Occupation**					0.207
	**Employee**	5(5.56%)	11(3.82%)	0(0%)	
	**Employer**	0(0%)	2(0.69%)	0(0%)	
	**Engineer**	1(1.11%)	5(1.74%)	0(0%)	
	**Farmer**	1(1.11%)	4(1.39%)	0(0%)	
	**Military**	6(6.67%)	15(5.21%)	0(0%)	
	**other**	1(1.11%)	23(7.99%)	2(10%)	
	**Teacher**	14(15.56%)	23(7.99%)	4(20%)	
	**Unemployed**	63(70%)	209(72.57%)	14(70%)	
**Type of Cancer**					0.255
	**Acute leukemia**	0(0%)	2(0.69%)	0(0%)	
	**Acute myeloid leukemia**	0(0%)	3(1.04%)	0(0%)	
	**Bladder**	3(3.33%)	6(2.08%)	0(0%)	
	**Brain tumor**	0(0%)	3(1.04%)	0(0%)	
	**Breast**	53(58.89%)	113(39.24%)	17(85%)	
	**Cholangiocarcinoma**	0(0%)	2(0.69%)	0(0%)	
	**Chronic leukemia**	1(1.11%)	2(0.69%)	0(0%)	
	**Chronic myeloid leukemia**	0(0%)	1(0.35%)	0(0%)	
	**Colon**	7(7.78%)	29(10.07%)	0(0%)	
	**Esophageal**	0(0%)	2(0.69%)	0(0%)	
	**Gastric**	5(5.56%)	16(5.56%)	0(0%)	
	**HCC**	2(2.22%)	3(1.04%)	0(0%)	
	**Larynx**	0(0%)	3(1.04%)	0(0%)	
	**Lung**	3(3.33%)	20(6.94%)	0(0%)	
	**Lymphoma**	2(2.22%)	5(1.74%)	1(5%)	
	**Neuroendocrine**	0(0%)	3(1.04%)	0(0%)	
	**Pancreas**	0(0%)	7(2.43%)	0(0%)	
	**Pharynx**	0(0%)	1(0.35%)	0(0%)	
	**Prostate**	2(2.22%)	24(8.33%)	0(0%)	
	**Rectum**	1(1.11%)	1(0.35%)	0(0%)	
	**Renal cell carcinoma**	1(1.11%)	7(2.43%)	0(0%)	
	**Salivary gland**	1(1.11%)	2(0.69%)	0(0%)	
	**Sarcoma**	0(0%)	7(2.43%)	0(0%)	
	**Tongue**	0(0%)	1(0.35%)	0(0%)	
	**Unknown**	0(0%)	3(1.04%)	0(0%)	
	**Uterus**	2(2.22%)	5(1.74%)	0(0%)	
	**Uveal melanoma**	1(1.11%)	0(0%)	0(0%)	
	**Cervix**	2(2.22%)	0(0%)	0(0%)	
	**Gallbladder**	2(2.22%)	1(0.35%)	0(0%)	
	**Ovarian**	2(2.22%)	12(4.17%)	1(5%)	
	**Squamous cell carcinoma**	0(0%)	3(1.04%)	0(0%)	
	**Testis**	0(0%)	1(0.35%)	1(5%)	
**Family History**					<0.0001
	**Aunt**	0(0%)	0(0%)	14(70%)	
	**Brother**	5(5.56%)	0(0%)	0(0%)	
	**Cousin**	0(0%)	0(0%)	5(25%)	
	**Father**	8(8.89%)	0(0%)	0(0%)	
	**Grandmother**	0(0%)	0(0%)	1(5%)	
	**Mother**	21(23.33%)	0(0%)	0(0%)	
	**No**	0(0%)	285(98.96%)	0(0%)	
	**Sister**	56(62.22%)	0(0%)	0(0%)	
	**Uncle**	0(0%)	3(1.04%)	0(0%)	
**Marriage**					0.048
	**Divorced**	0(0%)	2(0.69%)	0(0%)	
	**Married**	89(98.89%)	255(88.54%)	19(95%)	
	**Single**	1(1.11%)	31(10.76%)	1(5%)	
**Smoker**					0.688
	**No**	75(83.33%)	228(79.17%)	16(80%)	
	**Yes**	15(16.67%)	60(20.83%)	4(20%)	
**Alcohol**					0.856
	**No**	89(98.89%)	284(98.61%)	2(10%)	
	**Yes**	1(1.11%)	4(1.39%)	0(0%)	
**Dietary Habits**					0.206
	**Balanced**	82(91.11%)	268(93.06%)	16(80%)	
	**Mostly Meat**	4(4.44%)	21(7.29%)	3(15%)	
	**Mostly Vegetables**	4(4.44%)	6(2.08%)	1(5%)	
**BMI Category**					0.819
	**Underweight**	3(3.33%)	9(3.13%)	0(0%)	
	**Normal**	23(25.56%)	72(25%)	3(15%)	
	**Overweight**	31(34.44%)	105(36.46%)	8(40%)	
	**Obese I**	22(24.44%)	53(18.4%)	6(30%)	
	**Obese II**	9(10%)	31(10.76%)	2(10%)	
	**Obese III**	2(2.22%)	18(6.25%)	1(5%)	

**Table:** Table2. distribution of blood groups among categories of familial cancer

**Variable**	**First Degree (n=90)**	**None (n=288)**	**Second Degree (n=20)**	**Total (n=398)**	**Pearson χ² (df)**	**p-value**
**Blood Group**					15.6640 (14)	0.334
**A+**	27(30%)	100(34.72%)	7(35%)	134		
**A-**	3(3.33%)	6(2.08%)	1(5%)	10		
**AB+**	7(7.78%)	15(5.21%)	0(0%)	22		
**AB-**	1(1.11%)	3(1.04%)	1(5%)	5		
**B+**	17(18.89%)	61(21.18%)	4(20%)	82		
**B-**	1(1.11%)	4(1.39%)	0(0%)	5		
**O+**	30(33.33%)	98(34.03%)	6(30%)	134		
**O-**	4(4.44%)	1(0.35%)	1(5%)	6		

The Table-1 presents a detailed comparison of various demographic, socioeconomic, and clinical characteristics of cancer patients based on their family history of cancer in first-degree and second-degree relatives. The study includes three groups: patients with a family history of cancer in first-degree relatives (n=90), patients with no family history of cancer (n=288), and patients with a family history of cancer in second-degree relatives (n=20). The age distribution shows a significant trend (P=0.052) with the 38-47 and 48-57 age groups being the most represented in the first-degree and no family history groups, respectively. The 58-67 age group is more common in the first-degree group, while the 68-77 and 78-87 age groups are more prevalent in the no family history group. The sex distribution is significantly different (P=0.017) with a higher proportion of females in the first-degree and second-degree groups compared to the no family history group. The residence, educational level, and smoking and alcohol consumption habits do not show significant differences among the groups (P=0.268, P=0.614, P=0.688, and P=0.856, respectively). However, the socioeconomic state shows a trend towards significance (P=0.055) with a higher proportion of high socioeconomic status in the second-degree group and a higher proportion of middle socioeconomic status in the first-degree group.

The type of cancer also shows some differences among the groups, though not statistically significant (P=0.255). Breast cancer is more prevalent in the first-degree group (58.89%) and second-degree group (85%) compared to the no family history group (39.24%). Other cancers such as prostate, ovarian, and lymphoma is also more common in the first-degree group. The family history of cancer is highly significant (P<0.0001) with a majority of first-degree relatives (sisters, mothers, and brothers) and second-degree relatives (aunts and cousins) reported. The marital status is significantly different (P=0.048) with a higher proportion of married individuals in the first-degree and second-degree groups. Dietary habits show a trend (P=0.206) with a higher proportion of balanced diets in the no family history group and mostly meat diets in the second-degree group. The BMI category does not show significant differences (P=0.819) among the groups, with a similar distribution of underweight, normal, overweight, and obese categories.

The distribution of blood groups does not show statistically significant differences among the groups (P=0.334, df=14, Pearson χ² = 15.6640), Table-2. Specifically, the A+ blood group is the most common, with 30% in the first-degree group, 34.72% in the no family history group, and 35% in the second-degree group. The A- blood group is less common, with 3.33% in the first-degree group, 2.08% in the no family history group, and 5% in the second-degree group. The AB+ blood group is present in 7.78% of the first-degree group, 5.21% of the no family history group, and is absent in the second-degree group. The AB- blood group is also less common, with 1.11% in the first-degree group, 1.04% in the no family history group, and 5% in the second-degree group. The B+ blood group is found in 18.89% of the first-degree group, 21.18% of the no family history group, and 20% in the second-degree group. The B- blood group is present in 1.11% of the first-degree group, 1.39% of the no family history group, and is absent in the second-degree group. The O+ blood group is also common, with 33.33% in the first-degree group, 34.03% in the no family history group, and 30% in the second-degree group. The O- blood group is less common, with 4.44% in first-degree group, 0.35% in the no family history group, and 5% in the second-degree group. Despite these variations, the overall distribution of blood groups does not show a statistically significant difference among the groups.Top of Form

The provided data in Table-3 presents the results of a multinomial logistic regression analysis exploring the association between ABO/Rh blood groups and familial cancer history, adjusted for several demographic and lifestyle factors including age, sex, marital status, residence, education, socioeconomic status, occupation, type of cancer, smoking, alcohol consumption, dietary habits, and BMI. For first-degree relatives (n=90), the blood group O- shows a significant positive association with an odds ratio (OR) of 14.083 (95% CI: 1.412 to 140.447, P=0.024), indicating a higher likelihood of familial cancer history. Other blood groups (A-, AB+, AB-, B+, B-, O+) do not show significant associations, with p-values ranging from 0.542 to 0.944. For second-degree relatives (n=20), the blood group O- also shows a positive but non-significant association (OR: 9.9, 95% CI: 0.469 to 208.911, =0.141). Notably, some blood groups (AB+ and B- for second-degree relatives) have zero cases, leading to non-estimable (NE) confidence intervals. The overall findings suggest a potential link between the O- blood group and familial cancer history, particularly in first-degree relatives, while other blood groups do not demonstrate significant associations.

## Discussion

**Table:** Table3. Multinominal logistic regression between ABO/rh blood group and familial cancer history, adjusted for age, sex, marital status, residence, education, socioeconomic status, occupation, type of cancer, smoking, alcohol consumption, dietary habits, and BMI

	**OR**	**95% CI**		P-value
		**lower**	**upper**	
**First Degree (n=90)**				
**A-**	1.603	0.351	7.312	0.542
**AB+**	1.92	0.671	5.496	0.224
**AB-**	1.089	0.102	11.599	0.944
**B+**	1.128	0.553	2.304	0.74
**B-**	0.783	0.076	8.026	0.837
**O+**	1.194	0.644	2.214	0.574
**O-**	14.083	1.412	140.447	0.024
**Second Degree (n=20)**				
**A-**	1.592	0.141	17.995	0.707
**AB+**	0	0	NE	0.992
**AB-**	6.739	0.465	97.601	0.162
**B+**	0.986	0.253	3.839	0.984
**B-**	0	0	NE	0.996
**O+**	0.871	0.263	2.887	0.821
**O-**	9.9	0.469	208.911	0.141

Our study found that potential link between familial cancer and O negative blood group. But this was not seen in most other studies. Gates et al. (2012) did not found association of familial cancer with the blood group [13]; while their controls were healthy individuals. In another similar study in Iran, none of genetic factors of breast cancer like HER2 and Ki67 were related to blood groups [14]. However, the relationship between BRCA1 and blood groups remains unclear, with some studies suggesting that BRCA1 epimutation in blood may not be transmitted from mother to daughters and may be a consequence of environmental exposure [15]. But, in case of other cancers, there are some evidences matching our findings. The relationship between an oncogene named KRAS and blood group has been investigated in various studies, with some findings suggesting a potential association between the two [16]. For instance, a study on colorectal adenocarcinoma found that ABO/Rh blood groups were statistically significantly associated with the risk of CRC, although no relationship was found between K-ras status and ABO blood group and Rh factor [16].

A recent study explored the connection between pancreatic cancer risk and the genetic characteristics of first-degree relatives of individuals with the disease. The research analyzed data from over 23,000 relatives of 3,268 pancreatic cancer patients, taking into account the patients’ blood type and genetic mutations associated with cancer susceptibility. The results showed that relatives of patients with certain genetic mutations had a significantly higher risk of developing pancreatic cancer, with a nearly four-fold increased risk for those related to patients with non-O blood types and cancer-causing genetic mutations. Conversely, relatives of patients without such mutations had a lower, yet still elevated, risk of pancreatic cancer. The study’s findings suggest that considering both blood type and genetic mutation status can help estimate pancreatic cancer risk in first-degree relatives, with important implications for early detection and prevention strategies [17]. While our study and their study differ in focus and population, they both suggest a potential link between ABO blood group and cancer risk, with the Iraqi study highlighting the O-blood group as a potential risk factor for familial cancer.

A recent study examined the interaction between ABO blood groups and two important genes, FUT2 and FUT3, which determine secretor status and Lewis antigens, respectively. The study analyzed data from over 8,000 pancreatic cancer cases and 11,000 controls, and found that the increased risk associated with non-O blood groups was stronger in individuals with a specific genetic variant that determines secretor status, known as FUT2. In contrast, the study found no interaction between ABO blood groups and Lewis antigens, which are determined by the FUT3 gene. The results suggest that the relationship between ABO blood type and pancreatic cancer risk is modified by secretor status, but not by Lewis antigens, and highlight the importance of considering multiple genetic factors when assessing an individual’s risk of developing pancreatic cancer [18]. This shows that the association we found in our study must be further evaluated for each cancer type and based on interactions with different genes. In case of pathophysiology of this association, research has shown that changes in the structure and expression of ABO blood group antigens are associated with human cancer. Specifically, alterations in the A and H antigens, which are normally found on the surface of red blood cells, have been observed in cancer cells. These changes can affect the way cancer cells interact with their environment and may influence the progression and metastasis of the disease. For example, the A2 allele, which is associated with a weaker expression of the A antigen, has been found to be more common in certain types of cancer, suggesting a possible link between ABO blood type and cancer risk. Furthermore, the enzymes responsible for synthesizing the ABO antigens, such as the A enzyme, have been found to be altered in cancer cells, leading to changes in the expression of these antigens and potentially contributing to the development and progression of cancer [19].

This study has several limitations that need to be considered when interpreting the results. One of the major limitations is the relatively small sample size, particularly in the second-degree relatives’ group, which may not be representative of the larger population. Additionally, the study relied on self-reported data, which may be subject to recall bias and social desirability bias. The study also did not control for other potential confounding variables, such as genetic mutations, that may influence the relationship between ABO blood group and familial cancer occurrence. Furthermore, the study was conducted in a case only manner that considering the control healthy cases would increase the quality of evidence.

Further research is needed to fully understand the relationship between ABO blood group and familial cancer occurrence. Future studies should aim to recruit larger and more diverse samples, including participants from different cities and countries. It would also be beneficial to collect more detailed information on genetic mutations, lifestyle habits, and environmental exposures to control for potential confounding variables. Additionally, studies could investigate the potential mechanisms by which ABO blood group may influence cancer risk, such as differences in immune function or inflammation. Longitudinal studies could also be conducted to examine the incidence of cancer in individuals with different ABO blood groups over time. Moreover, research could focus on specific types of cancer, such as breast cancer, which was found to be more prevalent in the first-degree and second-degree groups in this study. By addressing these research gaps, we can gain a better understanding of the relationship between ABO blood group and familial cancer occurrence and potentially identify new strategies for cancer prevention and early detection.

## Conclusion

In conclusion, this study suggests a potential association between O- blood group and familial cancer occurrence in first-degree relatives, which warrants further investigation. The study’s findings show the importance of considering ABO blood group as a potential risk factor for familial cancer, particularly in individuals with a family history of cancer. While the study has several limitations, it contributes to the growing body of research on the relationship between blood type and cancer risk. Further research is needed to confirm these findings and to explore the underlying mechanisms by which ABO blood group may influence cancer risk. Ultimately, a better understanding of the relationship between ABO blood group and familial cancer occurrence could lead to the development of personalized cancer screening and prevention strategies, improving outcomes for individuals at high risk of developing cancer.

## Conflict of Interest

None.
